# “*As soon as I start trusting human beings, they disappoint me, and now I am going to get on an app that someone could hack. I really do not want to take that chance*”: barriers and facilitators to digital peer support implementation into community mental health centers

**DOI:** 10.3389/fdgth.2023.1130095

**Published:** 2023-07-20

**Authors:** Karen L. Fortuna, Shreya Divatia, Sudeep Neupane, Pamela Geiger, Andrew Bohm

**Affiliations:** ^1^ Department of Psychiatry, Dartmouth Centers for Health and Aging, Geisel School of Medicine, Lebanon, NH, United States; ^2^ Department of Public Health, Robbins College of Human Health and Sciences, Baylor University, Waco, TX, United States

**Keywords:** serious mental illness, implementation science, digital peer support, technology, mental health

## Abstract

**Background:**

Certified peer support specialists often use technologies such as smartphone applications to deliver digital peer support in community mental health centers. Certified peer support specialists are individuals with a mental health diagnosis, trained and accredited by their state to provide mental health support services. Digital peer support has shown promising evidence of promoting recovery, hope, social support, and medical and psychiatric self-management among patients with a diagnosis of a serious mental illness. Interest in digital peer support as part of the patient experience has grown. Understanding barriers and facilitators to the implementation process of digital peer support into community mental health centers is a critical next step to facilitate uptake.

**Methods:**

Semi-structured qualitative interviews were conducted with 27 patient participants (*N *= 17 persons with serious mental illness; *N *= 10 certified peer support specialists) from an urban community mental health center. Participants responded to open-ended questions on the barriers and facilitators of engaging with digital peer support technologies within community mental health centers. The interview guide and the responses were categorized according to the Consolidated Framework for Implementation Science Research (CFIR) constructs.

**Results:**

Nine *barriers* and two *facilitators* were identified for the implementation of digital peer support in community mental health centers. The overarching domains for the identified barriers included (1) intervention characteristics (i.e., adaptability, complexity, and cost), (2) inner settings (i.e., implementation climate, readiness for implementation, and access to knowledge and information), and (3) characteristics of individuals (i.e., knowledge and beliefs about the intervention and other personal attributes). The two facilitators identified included (1) intervention characteristics (i.e., relative advantage) and (2) outer setting (i.e., patient needs and resources).

**Conclusions:**

The identified barriers and facilitators represent a starting point for developing or modifying digital peer support technology requirements to ease implementation in community mental health centers. Building technology requirements and implementation processes based on these findings may facilitate uptake of digital peer support technologies by people with serious mental illness and certified peer support specialists in community mental health centers.

## Introduction

Certified peer support specialists may represent an effective solution for supporting recovery for people with serious mental illness (SMI) (1). SMI is defined as a mental illness resulting in serious functional impairment and limitations in one or more major life activities, (e.g., schizophrenia spectrum disorder, bipolar disorder, major depressive disorder) (2)_._ Certified peer support specialist support is defined as “social and/or emotional support that combines expertise from lived experience that is delivered with mutual agreement by persons who self-identify as having or had mental health as well as other social, psychological, and medical challenges to service users sharing similar challenges to bring about self-determined personal change to the service user” (3). In a previous systematic review, in-person peer support demonstrated reduction in self-stigma and inpatient service use, better treatment engagement, higher level of empowerment, higher levels of patient activation (perceived ability to self-manage health), and higher levels of hopefulness for recovery (4). The emergence of digital peer support (DPS) in 2005 (5) and the later expansion of digital peer support in 2019 due to the COVID-19 crisis has increased the reach of peer support services (5). Digital peer support is defined as “live or automated peer support services delivered through technology media” (6).

A 2019 systematic review found that digital peer support interventions showed promising evidence of increasing patient engagement in services as well as increases in empowerment, hope, quality of life, and medical and psychiatric self-management (7). However, most of the studies included in this systematic review did not examine implementation considerations of digital peer support. As such, while digital peer support technologies show burgeoning effectiveness, implementation in real-world settings outside of research settings is not known.

Understanding digital peer support implementation processes may facilitate uptake beyond clinical research settings. As such, this study aims to identify the barriers and facilitators of the implementation of digital peer support based on patients with SMI and certified peer support specialists’ perspectives.

## Methods

This study conducted semi-structured qualitative interviews with 27 participants (*N *= 17 persons with a serious mental illness and *N *= 10 certified peer support specialists) from an urban community mental health center. The data collection and research project were conducted following the ethical standards of the Institutional Review Board at Geisel School of Medicine at Dartmouth and with the 1964 Helsinki Declaration and its later amendments.

Agency staff reviewed clinical cases and identified patients that met study criteria. The criteria for the potential participant’s eligibility to participate in a semi-structured interview included the following: (1) participants will be adults aged 18 or older with a medical chart diagnosis of schizophrenia, schizoaffective disorder, bipolar disorder, or persistent major depressive disorder; (2) been enrolled in treatment at the community mental health center for at least 3 months; (3) able to speak and read English. Certified peer support specialists’ eligibility included the following: (1) certified peer support specialist in the state of Massachusetts (i.e., to qualify to be a certified peer support specialist, a person must self-report any mental health diagnosis, be in active treatment, and complete an 80-hour training that includes classes, small group activities, and homework on fundamentals of peer support, cross-cultural partnering, and human experience language. All certified peer support specialists must pass a written examination to become a Massachusetts certified peer support specialist); (2) speak and read English; and (3) must provide voluntary informed consent for participation in the study.

### Patient with SMI recruitment

Agency staff in a community mental health center discussed study details with potential participants with SMI using a standard script. If interested in participation, agency staff scheduled the screening, a written informed consent, and an interview with a research assistant trained in qualitative interviewing.

### Certified peer support specialist recruitment

Agency staff also approached certified peer support specialists within the same agency to discuss the study to assess interest in participating. Certified peer support specialists underwent a one-time screening and completed a written informed consent prior to semi-structured interviews with research staff.

### Interview guide development

The interview guide was co-produced with two certified peer support specialists (not interviewed for this study) using the Peer and Academic Model of Community Engagement (8). The interview guide was developed based on the Consolidated Framework for Implementation Science Research [CFIR) (9)]. CFIR is a conceptual framework that was developed to guide the systematic assessment of multilevel implementation contexts to identify factors that might influence intervention implementation and effectiveness (9). CFIR is composed of 38 constructs divided across five domains. Three domains of interest were chosen for evaluation in this study based on the expected barriers and facilitators. These domain themes included the following: (1) intervention characteristics (i.e., adaptability, complexity, and cost), (2) inner settings (i.e., implementation climate, readiness for implementation, and access to knowledge and information), and (3) characteristics of individuals (i.e., knowledge and beliefs about the intervention and other personal attributes). The interview guide included six broad questions and probes, as follows: (1) *are you familiar with technology such as digital peer support that can help people with SMI?*; (2) *do you think a digital peer support intervention would work in a community mental health center?*; (3) *do you think a digital peer support intervention would lead to improved outcomes for patients with SMI?;* (4) *what is new and interesting about digital peer support intervention?;* (5) *why would a digital peer support intervention work*?; and (6) *why wouldn’t a digital peer support intervention work? (Probe: technology is isolating, time pressure, lack of resources such as money to purchase a phone or data plan, insufficient materials, organizational constraints, insufficient support, lack of reimbursement, patients won’t want to be involved*).

### Interview process

Meeting with study staff occurred onsite, in-person at the community mental health center in a private room. Interview durations ranged from approximately half an hour to one-hour. All interviews were audio-recorded. Participants were compensated with $30 for participation. Qualitative interviews were audio recorded and transcribed. Qualitative interviews were conducted until we reached the saturation of data [i.e., saturation means that sampling more data will not lead to more information related to research questions (10)]. Member checking was employed to assess the validity of findings. Member checking is a technique for exploring the credibility of results where the data or results are returned to participants to check for accuracy and resonance with their experiences (10).

The sample consisted of 17 persons with SMI and 10 certified peer support specialists. The 17 patient participants had a mean age of 51 years and were primarily male (*n* = 12, 70.6%), white (*n* = 14, 82.4%), and included people with a self-report diagnosis of major depressive disorder (*n* = 5, 29.4%), schizophrenia, (*n* = 4, 23.5%), bipolar disorder (*n* = 4, 23.5%), or schizoaffective disorder (*n* = 3, 17.6%); one person did not report. Eight patient participants reported smartphone ownership (47%). Certified peer support specialists had a mean age of 40 years. All were female (*n* = 10, 100%) and white (*n* = 10, 100%). All certified peer support specialists had completed 80 h of certified peer support specialist training and were currently employed. All certified peer support specialists reported smartphone ownership.

### Data analysis

The data analysis was informed using a thematic analysis approach (11) (i.e., a method for identifying, studying, and communicating patterns in the data material). The first author (KF), third author (SN), and the fourth author (AB) read all the interview transcripts independently to familiarize themselves with the data. These authors assigned data-driven codes to segments of the text that represented relevant findings in the data aligned with the research purpose. The codes were collated and grouped into preliminary themes documenting reoccurring concepts or statements about the research subject. KF, AB, and SN met virtually and discussed the codes, their relations to the themes, as well as characteristics to and naming of each theme. Including two researchers in the coding process is considered important for validation purposes and for broadening the breadth and depth of the analysis (12). All authors agreed on the naming of the themes and placement of themes under CFIR domains. Data were triangulated to examine similarities and differences in responses from patients with SMI and certified peer support specialists.

## Results

Overall, 9 *barriers* and 2 *facilitators* to the implementation of digital peer support in community mental health centers were identified. The overarching domains for the identified barriers included (1) intervention characteristics (i.e., adaptability, complexity, and cost), (2) inner settings (i.e., implementation climate, readiness for implementation, and access to knowledge and information), and (3) characteristics of individuals (i.e., knowledge and beliefs about the intervention and other personal attributes). The domains for the facilitators included (1) outer setting (i.e., patient needs and resources) and (2) intervention characteristics (i.e., relative advantage). The results presented below include both barriers and facilitators to the implementation of digital peer support in community mental health centers as defined by certified peer support specialists and patients with SMI.

### Barriers to the implementation of digital peer support in community mental health centers

The barriers identified included three overarching themes (1) intervention characteristics (i.e., adaptability, complexity, and cost), (2) inner settings (i.e., implementation climate, readiness for implementation, and access to knowledge and information), and (3) characteristics of individuals (i.e., knowledge and beliefs about the intervention and other personal attributes).

#### Intervention characteristics

##### Adaptability

The most mentioned theme among the barriers to implementation included patients’ and certified peer support specialists’ views that the digital peer support technology should be adaptable to the needs of older patients (14/51.9%). Damschroder et al. defined “adaptability” as “the degree to which an intervention can be adapted, tailored, refined, or reinvented to meet local needs” (9). Patients desired an app able to be adapted to individual needs, including training for people with SMI to use the technology, and age-specific technology features (e.g., option to increase text size). For example, one patient participant reported, “*older generation does not necessarily like technology, and if the app is simple and easy to get into, that would help*.” A certified peer support specialist said, “*it might be difficult to implement with older clients who are not interested or savvy with technology*”.

##### Complexity

The second most prevalent theme among the barriers was that complex technologies would be a barrier to digital peer support engagement (6/22.2%).

Damschroder et al. defined “complexity” as the “perceived difficulty of the intervention, reflected by duration, scope, radicalness, disruptiveness, centrality, and intricacy and number of steps required to implement” (9). Patient participants reflected upon the challenges in using technology for persons with mental illnesses. For example, patients talked about “*difficulty understanding sentences*,” “*having to type*,” and “*overwhelming information*.” Participants also stated that technologies should be designed to fit everyone.

##### Cost

Participants with SMI and certified peer support specialists reported concern about the cost of implementing digital peer support interventions (5/18.5%). Damschroder et al. defined “cost” as “costs of the intervention and costs associated with implementing the intervention including investment, supply, and opportunity costs” (9). The cost of data plans, including the cost of the technologies such as mobile phones concerned both patient participants and certified peer support specialists. Both patient participants and certified peer support specialists mentioned that affordability of the digital peer support technology would be a barrier to use. For example, a patient explained, “*it could be expensive to own a smartphone and like the cost could deter people from wanting to get a smartphone*.”

### Inner setting

#### Implementation climate

The most prevalent theme in this domain was compatibility, including concerns about whether the implementation of the digital peer support technology would be compatible with patients’ real-world environment (9/33.3%). Damschroder et al. defined “compatibility” as “the degree of tangible fit between meaning and values attached to the intervention by involved individuals, how those align with individuals’ own norms, values, and perceived risks and needs, and how the intervention fits with existing workflows and systems” (9). Patients with SMI stated that concerns about privacy and security were barriers to the use of digital peer support technology. One person with SMI said, “*as soon as I start trusting human beings, they disappoint me, and now I am going to get on an app that someone could hack. I really do not want to take that chance*.” [emphasis added]. Another patient participant said, “*it should only be a supplement for therapy, they want confidentiality, and they want to know their personal information is safe*”.

#### Networks and communications

The second most prevalent theme in the Inner Setting domain related to the quality of communications within the organization (8/29.6%). Damschroder et al. defined “networks and communications” as “the nature and quality of webs of social networks and the nature and quality of formal and informal communications within an organization” (9). One certified peer support specialist saw technology as a place for self-expression and another said it could lead to more open communication between patient and clinician or certified peer support specialist. A certified peer support specialist reported that texting on a digital peer support mobile application would make it easier to connect when it is difficult to communicate via the phone. In this domain, Network and Communications, a certified peer support specialist also stated the need to consider preferences in communication, “*I just, I think that some people could say that it could take away from kind of like genuineness if there is like a tool in the middle…[emphasis added] but for some people it might be really great*.”

#### Readiness for implementation

##### Available resources

Patient participants and certified peer support specialists recommended tangible and intangible resources to attract people to use digital peer support services (8/29.6%). Damschroder et al. defined “available resources” as “the level of resources dedicated for implementation and on-going operations, including money, training, education, physical space, and time” (9). Certified peer support specialists believed that the patients would need device training, access to compatible mobile phones, and peer support services to utilize a digital peer support application. A certified peer support specialist reported “*a lot of people that we [peer specialists] service do not have the technology even to download an app*.” A certified peer support specialist said, “*you’d have to show them initially and show them how useful it is*.” A certified peer support specialist commented that “the more resources the better” and indicated a preference for an app that would “*put all the resources in one place for meditation, coping skills and support groups*”.

##### Access to knowledge and information

Patient participants and certified peer support specialists indicated that readiness for implementation of a digital peer support intervention would differ based on the information available regarding the application (5/18.5%). Damschroder et al. defined “access to knowledge and information” as the “ease of access to digestible information and knowledge about the intervention and how to incorporate it into work tasks” (9). Respondents indicated SMI are different mental illness and each person has different expectations regarding information and knowledge. This applies to the information needed to use the app (i.e., usability) as well as the information within the app (i.e., content related to specific symptoms or diagnoses).

### Characteristics of individuals

#### Knowledge and beliefs about the intervention

The most prevalent theme in this domain were the intrapersonal characteristics and attitudes towards implementation of digital peer support for the treatment of serious mental illness (17/63%). Damschroder et al. defined “knowledge and beliefs about the intervention” as “individual attitudes toward and value placed on the intervention as well as familiarity with facts, truths, and principles related to the intervention” (9). Many participants believed that technology would dilute the human connections that are significant in supporting people with mental health challenges. A patient stated, “*there are moments when you need to see that person face to face because you have so much on your mind that you cannot even concentrate even texting it*.”

#### Other personal attributes

An emerging theme in this domain included a person’s lateral capabilities (7/27, 25.9%). Damschroder et al. defined “other personal attributes” as a broad construct to include other personality traits such as tolerance of ambiguity, intellectual ability, motivation, values, competence, capacity, and learning style (9). For example, a patient indicated, “*I think if the specialist is giving you too much information at once, it is going to overwhelm you” and “they [patient] want as less people as possible to know about their condition*.”

### Facilitators to implement digital peer support in community mental health centers

The two facilitators identified included (1) outer setting (i.e., patient needs and resources) and (2) intervention characteristics (i.e., relative advantage).

#### Outer setting

##### Patient needs and resources

The most prevalent theme across all the domains represented how digital peer support can address patients’ needs in their mental health care (19/70.4%). Damschroder et al. defined “patient needs and resources” as the extent to which patient needs, as well as barriers and facilitators to meet those needs, are accurately known and prioritized by the organization (9). People with SMI emphasized that although they would prefer human interaction, digital peer support mobile applications would give access to a live or on-demand service at any time or in any place. Certified peer support specialists expressed that digital peer support mobile applications may help direct people with SMI to resources. In addition, a certified peer support specialist responded that “if they [people with serious mental illness] wanted to contact me by that app, that would be helpful.” When the interviewer asked how a mobile application could help the certified peer support specialist be more supportive in helping people manage their conditions, they responded, “*… more communication … making sure that they’re up to date with their medication.*”

#### Intervention characteristics

##### Relative advantage

A dominant theme in this domain included patients’ and certified peer support specialists’ views on the benefits of implementing digital peer support compared to in-person meetings with certified peer support specialists or a clinician (17/63%). Damschroder et al. defined “relative advantage” as the stakeholders’ perception of the advantage of implementing the intervention vs. an alternative solution (9). For example, a patient reported “*the app will always be there*” and a peer support specialist reported that a digital peer support tool would be “*easier for peer specialists.*” This peer support specialist had previous training in the use of technological interventions. Trust is mentioned as a facilitator when discussing the technology, because it “*would be delivered by someone you trust … peer support specialists have more empathy due to their lived experiences* [compared to clinicians]”. Another certified peer support specialist reported technology “*can bridge it [a person] until they get … support from their team…that would be useful*”. A patient reported the “*possibility of being able to track wellness and work in conjunction with your peer support person*” as a benefit to engagement. A certified peer support specialist also suggests that digital peer support “*would lead to improved outcomes for overall wellness and increased independence in clients for self-managing their conditions on their own during times when their peer isn’t available*”.

## Discussion

This study aimed to understand the barriers and facilitators to the implementation of digital peer support into community mental health centers. Patients with SMI have unique needs regarding training and access to digital peer support technologies and data plans. Face-to-face interaction is a preferable method for communication; however, both groups acknowledge the benefit and flexibility that utilizing digital peer support as an augmentative tool to clinical treatment. In addition, the age group and the diverse mental illness diagnoses of people with SMI concerned both the patients and certified peer support specialists regarding the adaptability of the technology and recommended personalizing technology to their needs.

Both patients and certified peer support specialists require resources to utilize digital peer services. The lack of smartphone ownership, technology training, data plan cost, and cost concerns hinder patients’ engagement to the digital peer support service. Training can partially offset these barriers. For example, lack of smartphone access due to cost can be addressed by training agency staff and peer support specialists about the availability of free or low-cost access programs like Safelink, which offers free smartphones and data plans to people who qualify for Medicaid reimbursement. Additionally, training on how to use technologies for both patients and certified peer support specialists may support user confidence and increase the uptake of technologies.

Patients and certified peer support specialists noted the value of human connection as the preferred method of interaction. As such, digital technologies that integrate automated, non-live interactions with digital peer support technologies such as chatbots may consider opportunities for live interactions with certified peer support specialists to increase acceptability and potential engagement in technologies. Both groups indicated that a benefit of digital peer support technologies as compared to live interaction was that the technologies could be accessed by the patient on-demand and as-needed without requiring a certified peer support specialist or a clinician to be available. Community mental health centers may consider multiple pathways to receive care, possibly through a hybrid technology and in-person model of care, or by encouraging use of the technologies for asynchronous support outside of normal operating hours.

Patients and certified peer support specialists acknowledged that digital peer support is a tool to enhance support outside of clinical environments. Younger adults may prefer to use technology to access digital peer support. Community mental health centers may consider conveying the purpose and the utilization of digital peer support technologies to address concerns that might hinder acceptance of the intervention, such as privacy, security, and confidentiality issues. Decision-support tools for certified peer support specialists and patients that address these issues through conversation and education may support engagement and transparency. Of note, technologies that sell data to third parties or require a high level of monitoring (i.e., ingestibles, wearables or analytical techniques such as digital phenotyping) may not be acceptable with this vulnerable population.

Patients and peer support specialists were concerned about age-related changes that may impact engagement (e.g., changes to eyesight may require an option to enlarge text) and technologies focusing on transdiagnostics without personalization to a patient’s needs. Digital peer support technologies should consider the nature of SMI and related variabilities in patients’ needs and symptoms (e.g., impairment in executive functioning). As such, digital peer support technologies could offer flexibility and customization to address patients’ specific needs related to SMI, cognitive deficits, and age-related preferences. The integration of precision medicine in digital peer support technologies may be a new area of innovation to enhance current digital peer support technologies.

The study has limitations and findings should be interpreted with caution. First, the patients and certified peer support specialists belonged to only one community mental health center. This may limit the generalizability of the results to other settings. Of note, this study only included patients and certified peer support specialists. Including additional staff members such as social workers, case managers, IT staff and also administrative leaders could expand our knowledge of the barriers and facilitators to the implementation of digital peer support. These groups have decision-making authority within community mental health centers can enhance our understanding of implementation decisions. Finally, all the certified peer support specialists and most of the patient participants in this study were White. Further research should be conducted to investigate potential sociocultural differences in barriers and facilitators to the use of digital peer support technologies. For example, African Americans have higher rates of distrust in the medical system which may amplify the concerns about privacy and security that were identified in this study (13). Nonetheless, this study provides an *initial* understanding from end-users of digital peer support.

## Conclusions

These findings enrich our understanding of the perspectives of patients and certified peer support specialists and may support the implementation of digital peer support technologies in community mental health centers. Training for certified peer support specialists, patients, and agency staff (including technology professionals) is an important area of current focus. Selecting digital peer support technologies that integrate live (not chatbots) certified peer support specialists and features to support older adult uptake of technology may support uptake. This population may be more sensitive to concerns regarding privacy, security, and confidentiality for peer support technologies. Therefore, invasive technologies such as ingestibles, wearables or those that include analytical techniques such as digital phenotyping may not be acceptable. Future technology development may consider precision medicine in technologies to support patient needs and preferences.

## Data Availability

The raw data supporting the conclusions of this article will be made available by the authors, without undue reservation.
